# Resistant starch and exercise independently attenuate weight regain on a high fat diet in a rat model of obesity

**DOI:** 10.1186/1743-7075-8-49

**Published:** 2011-07-07

**Authors:** Janine A Higgins, Matthew R Jackman, Ian L Brown, Ginger C Johnson, Amy Steig, Holly R Wyatt, James O Hill, Paul S MacLean

**Affiliations:** 1 Center for Human Nutrition, Anschutz Medical Campus, University of Colorado Denver; 13001 E 17th Place, Campus Box C263, Aurora, CO 80045, USA; 2 Department of Medicine, Division of Endocrinology, Diabetes and Metabolism, Anschutz Medical Campus, University of Colorado Denver; 12800 East 19th Avenue, Mailstop F8305, Aurora, CO, 80045, USA; 3 Department of Pediatrics, Anschutz Medical Campus, University of Colorado Denver; Children's Hospital Colorado, 13123 E 16th Avenue, B218, Aurora, CO. 80045, USA

## Abstract

**Background:**

Long-term weight reduction remains elusive for many obese individuals. Resistant starch (RS) and exercise may be useful for weight maintenance. The effects of RS, with or without exercise, on weight regain was examined during relapse to obesity on a high carbohydrate, high fat (HC/HF) diet.

**Methods:**

Obesity-prone rats were fed *ad libitum *for 16 weeks then weight reduced on a low fat diet to induce a 17% body weight loss (weight reduced rats). Weight reduced rats were maintained on an energy-restricted low fat diet for 18 weeks, with or without a daily bout of treadmill exercise. Rats were then allowed free access to HC/HF diet containing low (0.3%) or high (5.9%) levels of RS. Weight regain, energy balance, body composition, adipocyte cellularity, and fuel utilization were monitored as rats relapsed to obesity and surpassed their original, obese weight.

**Results:**

Both RS and exercise independently attenuated weight regain by reducing the energy gap between the drive to eat and suppressed energy requirements. Exercise attenuated the deposition of lean mass during relapse, whereas its combination with RS sustained lean mass accrual as body weight returned. Early in relapse, RS lowered insulin levels and reduced the deposition of fat in subcutaneous adipose tissue. Exercise cessation at five weeks of relapse led to increased weight gain, body fat, subcutaneous adipocytes, and decreased lean mass; all detrimental consequences to overall metabolic health.

**Conclusions:**

These data are the first to show the complimentary effects of dietary RS and regular exercise in countering the metabolic drive to regain weight following weight loss and suggest that exercise cessation, in the context of relapse on a HC/HF diet, may have dire metabolic consequences.

## Background

Weight regain occurs frequently in individuals who have lost weight [1]. Substantial evidence indicates that biological adaptations to weight loss contribute to weight regain. Reductions in leptin, insulin [2,3], and other neural, nutrient, and endocrine signals, convey an "energy deficit" signal to energy balance control centers within the brain resulting in an increased drive to eat [4] and suppressed energy expenditure [5-7]. Studies in obese rodent models of weight regain indicate that this large energy gap between elevated energy intake and suppressed energy requirements does not dissipate and may even increase with time in weight maintenance [8]. Peripherally, metabolic regulation improves and primes tissues to store any caloric excess in an energetically efficient manner [9,10]. Animal studies also show that the total number of cells increases in abdominal fat pads early in relapse, reflecting an increase in the number of very small adipocytes [10]. The increase in the total number of cells in these fat pads persists after the relapse to obesity, with the animals gaining more weight than they originally lost [11,12]. This hypercellularity after weight loss has been observed in some clinical studies [2], and it may facilitate both repletion and expansion of adipose tissue storage capacity. Together, the increase in energy intake and suppressed energy requirements promote the energy imbalance required for weight regain, and, when overfeeding occurs, the peripheral adaptations ensure rapid clearance of ingested nutrients, energetically efficient adipose repletion, and continued signals to the brain that perpetuate the energy imbalance until the lost weight returns.

Exercise attenuates weight regain after weight loss from obesity in both humans [13,14] and rodents [12,15]. The benefits of exercise have been attributed, at least in part, to countering the biological adaptations that drive weight regain [12,15]. Daily treadmill exercise in formerly-obese rodents reduces the rate of regain early in relapse and lowers the steady state weight achieved at the end of relapse [12]. Food intake is dramatically reduced such that *ad libitum *energy intake is closer to matching energy requirements. In the periphery, exercise promotes fat oxidation and prevents the relapse-induced hypercellularity in abdominal fat pads [12]. Because exercise affects a number of components in the homeostatic system controlling body weight, it is not surprising that the vast majority of individuals who are successful at keeping weight off long-term include regular, vigorous exercise in their weight maintenance program [16].

More generally, compliance with exercise prescriptions is relatively poor [17], and changes in diet are only transiently maintained [18]. Therefore, additional interventions that complement the benefits of exercise or impart benefit in the absence of exercise are sorely needed. Dietary resistant starch (RS) is one such strategy that may facilitate long-term weight reduction. RS is any starch or starch by-products that are not fully digested and absorbed in the upper digestive tract and pass to the large intestine, where they can be fermented by microbiota [19,20]. RS is found naturally in under-ripe bananas (green at the ends), plantains, beans and legumes, rolled oats, corn flakes, puffed wheat cereal, and corn tortillas. It is also prevalent in cooked then cooled starchy foods such as potatoes, rice, and pasta. So, RS intake can be boosted by eating potato salad rather than baked potatoes or pasta salad rather than hot pasta dishes. The average RS intake in US adults is about 5 g/d (4.4 g/d for women, 5.9 g/d for men) [21].

RS reduces postprandial glycemia and insulinemia [22,23], minimizes bone mass loss during weight loss/cycling [24], enhances secretion of gut-derived satiety signals [25,26], and promotes a catabolic profile in the arcuate nucleus of the hypothalamus [27]. Additionally, RS increases fat oxidation [28,29], reduces adipocyte size, and lowers the lipogenic capacity of adipose tissue [30]. Given these beneficial effects, we hypothesized that dietary RS would facilitate weight maintenance after weight loss and produce a more favorable body composition relative to a digestible starch diet. In addition, we hypothesized that the coordinated impact of both dietary RS and regular exercise would be more potent than either intervention alone.

Dietary RS, alone or in combination with exercise, has not been studied for its impact on the biological drive to regain lost weight. Neither intervention has been studied during relapse on a high fat diet, which would reflect both the failure to restrict energy intake and reversion to more obesogenic, high fat, high simple carbohydrate, foods. While this metabolic context may be common in humans, prospective clinical studies of relapse on a high fat diet would be unethical because of the known detrimental metabolic consequences of this diet. So, we employed a well characterized postobese rodent model of weight regain [8,10-12,31] to examine the effect of RS and exercise on the biological drive to regain weight on a high fat diet following a period of weight loss and maintenance. This paradigm was utilized to test the hypothesis that RS and exercise would independently and synergistically decrease the rate of weight regain and lower the overall defended body weight as the animals relapsed from weight maintenance to obesity on an obesogenic diet.

## Methods

### Animals

Male Wistar rats (n = 108, 125-150 g) were purchased from Charles River Laboratories (Wilmington, MA) and individually housed in the University of Colorado Denver (UCD) Center for Human Nutrition Satellite Facility (22-24°C; 12:12-h light-dark cycle; 3 pm lights out) with free access to water. Procedures were approved by the University of Colorado Denver Institutional Animal Care and Use Committee.

### Study Design

After acclimating to the facility for one week, rats were selected for their polygenic predisposition to become obese at 10 weeks of age according to their response to one week on a diet high in fat (46% kcal fat, Research Diets, Inc., NJ; RD# 12344.), as we have done previously [8,10-12,31]. The top tertile of weight gainers, designated as obesity-prone, continued to feed on this high fat diet with limited physical activity (individually housed in 22 × 22 × 18 cm hanging cages) for 16 weeks. At this point, rats are fully mature, as lean mass accrual tapers off and body weight gain declines [8,11,12]. Subsequent changes in weight primarily reflect the progression of obesity rather than growth.

**Figure 1:**
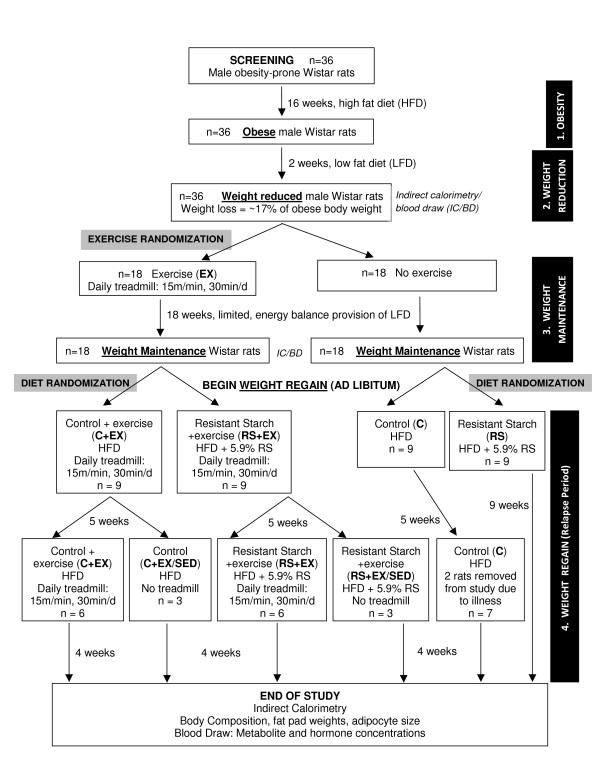
**Schematic for overall study design**.

Obese rats were placed on a calorie-restricted low fat diet (LFD, 12% kcal fat, Research Diets, Inc., NJ; RD# 11724) for two weeks, which reduced body weight by ~17%. Energy intake during the weight loss period was restricted to ~60% of the obese energy intake with daily monitoring and adjustment of diet provision throughout the 14 day period to ensure a negative energy balance. As in our previous studies [12], weight-reduced rats were stratified according to weight and weight loss, and divided into sedentary (SED) and exercise (EX) groups for the duration of the study. For 18 weeks, rats were maintained at this reduced weight with a limited provision of LFD under either sedentary conditions or with a daily regimen of treadmill exercise (Figure 1). The amount of food offered to each rat during weight maintenance was initially matched to energy expenditure measurements obtained during indirect calorimetry on days 0 and 1. Rats were weighed daily and food was added or subtracted from the daily ration if rats lost or gained weight, respectively.

EX and SED rats were then further divided into four groups that would relapse to obesity on a diet high in carbohydrate (40% kcal) and fat (40% kcal) (HC/HF diet) with either 0.3% (control, C) or 5.9% (RS) kcal resistant starch (Figure 1). RS was included at the dose of 5.9% energy as this value has been previously reported to increase fat oxidation in healthy humans [28]. Furthermore, this is a dose that is within the range of normal human intake [21]. Diets were matched for macronutrient and micronutrient content. Amioca (low amylose, unmodified waxy maize starch) and Hi-Maize 958 (unmodified high amylose maize starch, Class VIII) were used to enriched the diets with digestible starch or RS (type RS2, Table 1). Starches were analyzed for resistant starch content according to the AOAC 2002.02 method (Megazyme International Ireland Ltd.). The two groups that remained sedentary on these diets (C, RS) and the two groups that exercised on these diets (C+EX, RS+EX) were given an energy balance provision of their respective diets on the final day of weight maintenance to familiarize rats with their new diets (Day 0 of relapse). For the remainder of the study (days 1-65), rats were given *ad libitum *access to these diets while they continued their SED or EX regimens. During the last four weeks of relapse, two sedentary animals from the C group became ill and had to be removed from the study early. One animal developed weakness in the hind limbs and the other began eating less which caused rapid weight loss. These conditions do not seem to be related to the study intervention but both rats were euthanized to prevent suffering.

**Table 1:** Composition of the Relapse Diets.

	C		RS	
**MACRONUTRIENTS**	**gm%**	***kcal%***	**gm%**	***kcal%***
**PROTEIN**	24	*21*	24	*21*
**CARBOHYDRATE**	45	*39*	45	*39*
**FAT**	21	*40*	21	*40*

**TOTAL**	90	*100*	90	*100*

**Density (kcal/g)**	4.7		4.7	

**FIBER COMPONENTS**	**gm%**	***kcal%***	**gm%**	***kcal%***
**INSOLUBLE FIBER**	2.4%	0.0%	2.4%	0.0%
RS***	0.3%	*0.3%*	6.9%	*5.9%*

**TOTAL FIBER**	2.7%	*0.3%*	9.3%	*5.9%*

**INGREDIENT**	**gm**	***kcal***	**gm**	***kcal***
**PROTEIN (total)**	**203**	**812**	**203**	**812**

**Casein**	200	*800*	200	*800*
**DL-Methionine**	3	*12*	3	*12*
**CHO (total)**	**390**	**1480**	**390**	**1480**

**Maltodextrin**	150	*600*	150	*600*
**Sucrose**	87	*348*	87	*348*
Amioca *	133	*532*	0	*0*
Digestible Starch	130	*521*	0	*0*
Resistant Starch	3	*11*	0	*0*
Hi-maize 958 **	0	*0*	133	*532*
Digestible Starch	0	*0*	75	*300*
Resistant Starch	0	*0*	58	*232*
**Cellulose, BW200**	20	*0*	20	*0*
**FAT (total)**	**200**	***1575***	**200**	***1575***

**Corn Oil**	45	*405*	45	*405*
**Coconut Oil, Hydr**.	130	*1170*	130	*1170*
**OTHER (total)**	**47.1**	**40**	**47.1**	**40**
**Mineral-S10001**	35	*0*	35	*0*
**Vitamin-V10001**	10	*40*	10	*40*
**Choline Bitartrate**	2	*0*	2	*0*
**FD&C Dye**	0.1	*0*	0.1	*0*

**TOTAL CONTENTS**	**840**	**3907**	**840**	**3907**

Diets Prepared by Research Diets, New Brunswick, NJ.

* low amylose unmodified waxy maize starch.

** unmodified high amylose maize starch, Class VIII

*** Resistant Starch, type 2 (RS2)

To place rat data in context with that from humans, the shorter lifespan of the rat (2 yrs vs 80 yrs) must be considered. Obesity-prone rats were selected after sexual maturity, while their rate of growth reflected that of adolescence in humans. The rats matured into fully-grown adults under obesogenic conditions for 16 additional weeks. Weight loss and regain, therefore, occur on a compacted time line relative to the human, and the beginning of relapse (at 44 weeks) would roughly translate to a human between the ages of 30 and 35. The progressive weight regain observed in rats over 8 to 9 weeks [8,11,12,31] is similar to that observed in humans over 3 to 5 years [1].

### Treadmill Exercise

During the weight loss period, rats were acclimated to the treadmill environment (5 min/day), and then the training protocol was initiated at the beginning of the weight maintenance phase (Figure 1). EX rats were acclimated to the regimen of daily exercise (15 m/min for 30 min/day on 6 days/week) by gradually ramping up the speed and time over the first 3 weeks of weight maintenance. Rats were motivated to complete their daily training by using one or more of the following stimuli: 1) positioning food pellets outside the head of the lane; 2) dangling a novel play item at the head of the lane; 3) shock from an electric grid at the rear of the lane (10 V, 0.5A, 0.75 hz); 3) application of a bristle brush to the feet on the rear grid; and/or 4) intermittent air puffs to the hind-quarters. The type and combination of motivation used varied within the same rat and between rats, depending on the response to the motivation. Rats were scored on a graded scale by the technician for their performance (1 = poor; 5 = outstanding), based upon the amount of oversight and encouragement required to ensure compliance. This regimen of exercise was maintained for the study's duration (including both the weight maintenance and relapse phases of the protocol). At the end of the weight maintenance phase of the study, rats were well adapted to the exercise program while on the energy restricted dietary regimen (Compliance Score = 4.3 ± 0.2).

### Exercise Cessation

Five weeks into the relapse period, two C+EX rats and one RS+EX began to exhibit progressively poor compliance to the exercise regimen as they regained the lost weight (Compliance Score = 2.9 ± 0.4). These rats had to be removed from the exercise program for humane reasons. Because compliance to exercise prescriptions in humans is often transient, we deemed it worthwhile to continue to follow these animals for the remainder of the relapse period. To balance the design, three C+EX and three RS+EX rats with the lowest compliance scores at that time were removed from the exercise program and monitored on their respective diets under sedentary conditions (EX/SED).

### Energy Balance

Energy intake was measured regularly in metabolic housing racks throughout the relapse period. A metabolic monitoring system with indirect calorimetry (Columbus Instruments, Columbus OH), urine collection, and 24 hr food intake, was used to assess energy balance and fuel utilization at days 0, 1, and 65 of the relapse period. A two-day lead-in period was used to acclimate the rats to the metabolic chambers. Additional measurements were obtained in EX/SED rats at day 35, as they transition off of their exercise program. Metabolic rate (MR) was calculated from gas exchange measurements acquired every 10 minutes over the 24 h monitoring period, using the Weir equation (MR = 3.941*vO_2 _+ 1.106*vCO_2 _- 2.17*N), as previously described [8,12,31]. Urea and creatine excretion over the 24 h monitoring period was used to estimate urinary nitrogen [8,31]. Respiratory exchange ratio (RER) was calculated as the ratio of CO_2 _production to O_2 _consumption (vCO_2_/vO_2_). The difference between protein intake and protein disappearance was calculated as protein balance. Energetics of the exercise bout was assessed on days 0, 1, and 65, in an exercise indirect calorimetry chamber [12]. Steady-state measurements of metabolic rate and substrate oxidation during exercise were included in the 24 h estimates of expenditure and substrate disappearance. Feces were weighed and collected for the analysis of fecal fat and energy content throughout the study. A Folch lipid extraction was performed [10] to determine fecal lipid content.

### Tissue Analyses

Body composition was determined by dual-energy x-ray absorptiometry [8], both before and after the relapse period. Blood was collected via tail snip under brief isoflurane anesthesia at the end of the light cycle on day 0, 1, and 65, and isolated plasma was stored at -80°C for analysis. After the 9 week relapse period, fat pads were excised and weighed. As in our previous studies of adipocyte cellularity [10-12], cells from each sample were stained for integrity (methylene blue) after a brief collagenase digestion. Adipocytes were immediately viewed and imaged by a blinded microscopist with a 0.01 mm stage micrometer using an Olympus Max U-CMAD3 microscope and a C-mounted Canon Power Shot G5 digital camera. Images were analyzed with Cell Counting Analysis Program (Mayo Clinic, Rochester, MN) to obtain the diameter of 150 to 200 cells from each sample. Cell size frequency distributions were generated and the average cell size was determined for each sample. The total cell number within the pad was calculated from the relative number of cells in each subfraction, the lipid content of that subfraction (estimated by volume), and the total pad weight. Humoral metabolites, hormones, and urinary corticosterone were measured as previously described [11,32].

### Statistical Analyses

Data were analyzed with SPSS statistical software (version 19.0) and are expressed as mean ± SEM. Lead-in characteristics of SED and EX rats were compared with an independent t-test. A repeated measures ANOVA model was used to examine the effects of diet, exercise, time, and their interaction, including only those animals that completed their treatment regimens (C, RS, C+EX, RS+EX). Planned comparisons of RS-fed rats to their respective C-fed counterparts were used to detect differences between groups where specified. To assess the impact of exercise cessation, a repeated measures ANOVA model was used to examine the effect of exercise (C, EX, EX/SED), time, and their interaction. For table 2, a two-way ANOVA was performed on pooled data from all RS vs C rats, such that the RS-all group consisted of RS and RS+EX rats which was compared to the C-all group which was comprised of the C and C+EX groups. This analysis was performed to directly test the overall effects of RS vs exercise on weight regain which was the primary objective of this study. For other analyses, the statistical approach is specified in the figure or table. In some cases, an analysis of covariance (ANCOVA) was used to examine differences that remained after adjusting for a relevant covariate. The dependence of the intervention on cell size frequency distributions was examined with Pearson's χ^2 ^analysis. Pearson's correlation coefficients were calculated to examine the relationships between specified variables. Statistical significance was assumed at p ≤ 0·05.

**Table 2:** End of Study Body Composition and Adipocyte Cellularity

	C	RS	C+EX	RS+EX	C+EX/SED	RS+EX/SED
n	7	9	6	6	3	3
Final Weight (g)	846 ± 26	805 ± 32	792 ± 43	777 ± 33	847 ± 19	856 ± 40
% Body Fat ^A^	39.0 ± 3.2	33.9 ± 4.1	42.1 ± 1.3	32.0 ± 3.2 *	53.0 ± 1.4	41.9 ± 0.4 *
Fat Free Mass (g) ^ABC^	514 ± 24	525 ± 23	459 ± 29	524 ± 17.7*	398 ± 18	497 ± 20.9*
Bone Calcium (g) ^A^	13.2 ± 0.6	14.2 ± 0.6	13.0 ± 0.9	14.8 ± 0.6 *	11.8 ± 0.7	13.2 ± 0.7
Bone Density (g/cm^3^) ^A^	0.330 ± 0.002	0.339 ± 0.003	0.333 ± 0.004	0.339 ± 0.003	0.339 ± 0.000	0.333 ± 0.003
Gatrocnemius (g)	3.1 ± 0.1	3.0 ± 0.1	3.1 ± 0.1	3.1 ± 0.1	2.7 ± 0.1	3.2 ± 0.2
Liver (g)	18.8 ± 0.5	20.1 ± 0.4	18.5 ± 1.2	19.6 ± 1.3	21.6 ± 1.3	19.8 ± 0.4
Fat Mass (g) ^ABC^	332 ± 33	280 ± 42	333 ± 19	252 ± 32.3*	449 ± 11	359 ± 19.6*
Mesenteric Fat (g) ^AB^	21.3 ± 1.6	15.5 ± 1.0 *	15.7 ± 2.0	14.2 ± 1.9	21.6 ± 1.5	16.3 ± 1.2 *
Cell Diameter (μm) ^AB^	93.1 ± 0.8	88.4 ± 0.7 *	86.9 ± 0.9	76.3 ± 0.8 *	102.4 ± 1.1	95.4 ± 1.1 *
Cell Number (x10^6 ^cells/depot)	42.3 ± 8.9	47.2 ± 7.2	47.9 ± 7.4	56.4 ± 8.9	39.2 ± 10.5	35.6 ± 10.5
Subcutaneous Fat (g) ^A^	246 ± 32	199 ± 37	251 ± 16	181 ± 27 *	350 ± 12	270 ± 14 *
Cell Diameter (μm) ^AB^	80.8 ± 1.1	76.9 ± 0.7 *	74.6 ± 0.9	68.9 ± 0.7 *	82.3 ± 1.0	76.3 ± 1.0 *
Cell Number (x10^6 ^cells/depot) ^B^	655 ± 110	704 ± 59	985 ± 89	966 ± 89	1111 ± 177	1149 ± 356
Retroperitoneal Fat (g)	41.7 ± 1.9	43.1 ± 5.2	44.3 ± 6.6	38.2 ± 4.2	49.3 ± 4.6	45.3 ± 1.9
Epidydimal Fat (g) ^B^	23.5 ± 0.9	22.2 ± 1.3	22.4 ± 2.0	18.5 ± 2.8	27.6 ± 2.3	27.7 ± 1.9

End of study characteristics were examined in a two-way ANOVA model for the effects of diet (C vs RS) and exercise (SED, EX, EX/SED).

A = effect of diet, p < 0.05; B = effect of exercise, p < 0.05; C = diet × exercise, p < 0.05.

* planned comparison, RS-fed significantly different from respective C-fed group, p < 0.05.

## Results

### Weight Reduction and Maintenance Characteristics

Energy balance, fuel utilization, and body composition characteristics of obese and weight reduced rats in this model have been reported elsewhere [8,10-12,31,33], and this cohort reflected these general characteristics. Obese rats prior to weight loss averaged 726 ± 9 g (~33% body fat; Table 3). In response to the calorie-restricted, weight loss intervention, rats lost an average of 17.0 ± 0.5% of body weight. Rats were maintained at this reduced weight with a limited provision of LFD under sedentary conditions (SED) or with a regimen of treadmill exercise (EX). At the end of weight maintenance, the SED and EX groups were similar in weight, total bone mass, bone density, fat free mass (FFM), fat mass (FM), and percent body fat (Table 3).

**Table 3:** Weight Reduction and Maintenance Characteristics.

	SED	EX
n	18	18
Peak Obesity Weight (g)	723 ± 14	728 ± 12
Weight Loss (g)	118 ± 5	129 ± 7
Weight Maintenance		
Weight (g)	605 ± 11	599 ± 11
Bone Calcium (g)	13.3 ± 0.3	13.1 ± 0.3
Bone Density (g/cm^3^)	0.334 ± 0.002	0.335 ± 0.002
Fat Free Mass (g)	469 ± 12	473 ± 11
Fat Mass (g)	146 ± 15	131 ± 13
% Body Fat	23.4 ± 2.2	21.4 ± 2.0

Lead-in characteristics were examined by ANOVA for the effect of exercise (SED vs EX).

### Weight Regain

**Figure 2:**
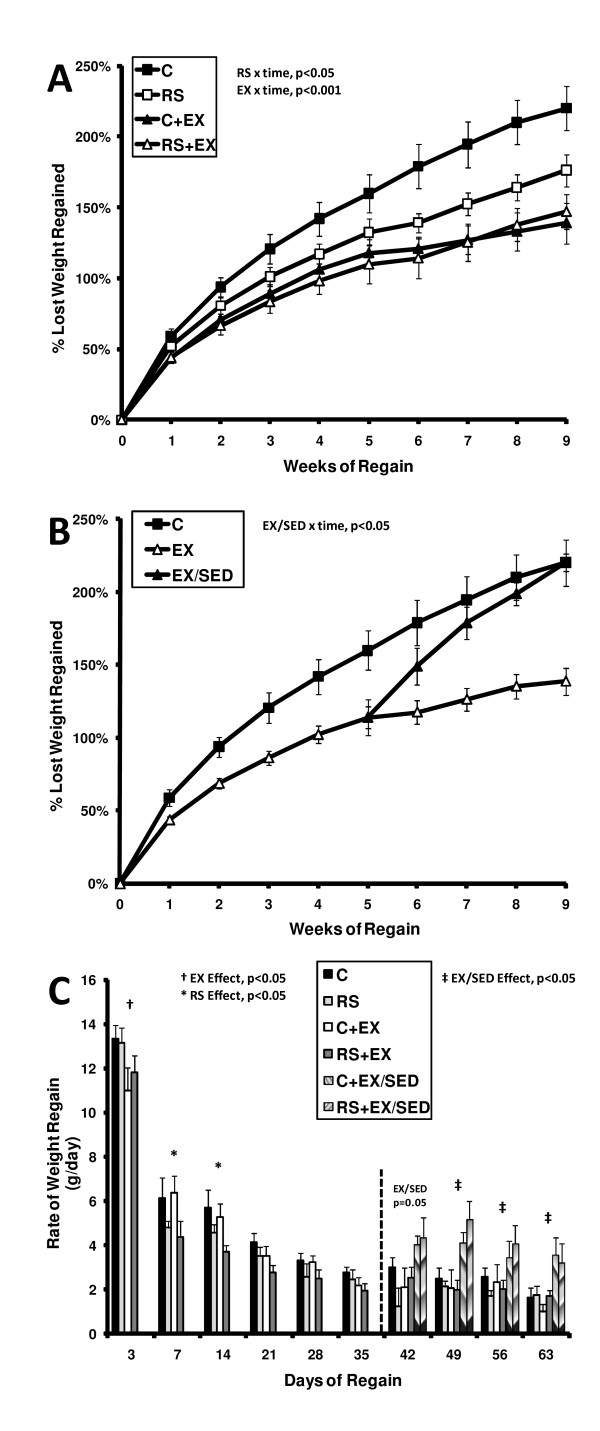
**Weight Regain After Weight Loss**. **(A) **The percent of lost weight that was regained during the 9 week period of relapse for C, RS, C+EX and RS+EX groups is shown. Data were examined in a repeated measures ANOVA model for an effect of diet (RS), exercise (EX), time, and their interaction. EX/SED rats were excluded from this analysis. **(B) **The percent of lost weight that was regained during the last 4 weeks of relapse is shown for C rats (n = 7), all rats that exercised (EX, n = 12), and all rats that exercised, but became sedentary at week 5 of relapse (EX/SED, n = 6). Data were examined in a repeated measures model for the effect of EX/SED (C vs EX vs EX/SED), time, and their interaction. RS rats were excluded from this analysis. **(C) **The rate of gain at regular intervals was examined to determine the stages of relapse during which the groups differed. Data during the first 5 weeks of relapse was examined in a two-way ANOVA for the effect of diet (RS), exercise (EX), and their interaction. Only data within the first two weeks exhibited a significant effect of exercise or diet. After week 5, a separate analysis of effect of EX/SED (C vs EX vs EX/SED) revealed a higher rate of gain in EX/SED than C and EX rats for the remainder of the study.

Both exercise and RS reduced the percentage of lost weight that was regained over the 9 week relapse period (RS p < 0.05, EX p < 0.001; Figure 2A). Based solely upon the percentage of lost weight regained, the combination of RS and exercise induced an effect greater than that of RS alone (p < 0.05), but not of exercise alone. In a subset of EX rats, the cessation of exercise at week five of relapse was followed by an increase in % of lost weight that was regained during the last four weeks of relapse (Figure 2B). By 9 weeks of relapse, EX/SED rats had regained weight so that their overall body weight was comparable to the C sedentary group (Figure 2B). To determine the temporal impact of the interventions, the rate of weight gain was examined at several intervals of the relapse process (Figure 2C). The rate of weight regain was reduced by exercise in the first few days of relapse and by RS from days 3 to 14 (Figure 2C).

### Body Composition

At the end of the study, body composition and tissue weights were recorded (Table 2). In an analysis that combined all animals, RS-all rats had significantly higher FFM (517 ± 15 vs 470 ± 16 g, p < 0.05), lower FM (280 ± 21 vs 351 ± 22 g, p < 0.05), and lower percent body fat (34.1 ± 2.1 vs 42.2 ± 2.2%, p < 0.01), compared to C-all rats. The impact of RS on fat accumulation was apparent in both mesenteric and subcutaneous adipose depots (Table 2). RS-all rats also had a higher total bone calcium content (14.2 ± 0.4 vs 12.9 ± 0.4 g, p < 0.05) and tended to have a higher bone mineral density (0.338 ± 0.002 vs 0.333 ± 0.002 g/cm^3^, p = 0.07) than C-all animals.

Exercise alone led to weight gain comprised entirely of fat (Table 2) such that the lower body weight of C+EX rats vs C rats at the end of the study was entirely due to a loss of FFM. However, adding RS led to higher FFM (C+EX vs RS+EX, p < 0.05) and lower FM (C-EX vs RS+EX, p < 0.05) accumulation during the relapse period. This beneficial effect of RS on the type of weight that was regained was also apparent in animals that stopped exercising at week 5 of relapse (Table 2, C+EX/SED vs RS+EX/SED, p < 0.05).

### Adipocyte Cellularity

**Figure 3:**
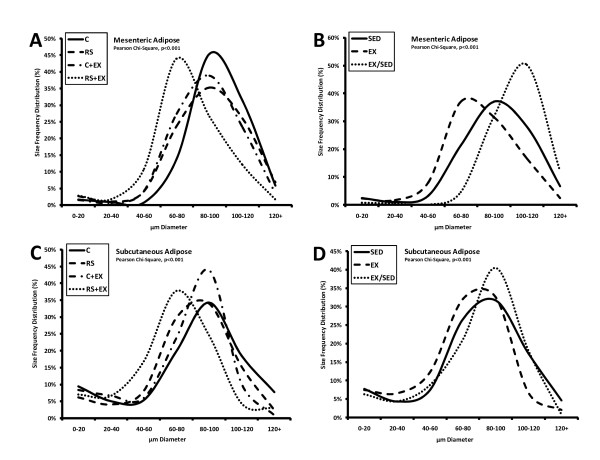
**Cell Size Frequency Distribution Profiles of Adipose Tissue**. Distribution of adipocyte size for mesenteric (**A-B**) and subcutaneous (**C-D**) adipose depots. The impact of dietary RS and exercise is shown in panels **A **and **C**, whereas the impact of exercise and exercise cessation for the two depots is shown in panels **B **and **D**. Profiles were created from the compiled cells of all animals in the groups (~150 cell/rat), stratified into 20 μm intervals. The profile shifts in mesenteric fat were accompanied by no change in total number of mesenteric adipocytes (Table 2). In contrast, exercise and exercise cessation were accompanied by a progressive increase in the number of adipocytes in subcutaneous adipose tissue (see Table 2). The dependence of the frequency distributions on the interventions was examined with Pearson's χ^2 ^analysis.

In mesenteric (one component of visceral) fat, variation in cell size, rather than cell number, accounted for differential fat pad weight between the groups (Table 2; Figure 3A). Exercise cessation led to a higher average size without affecting cell number in mesenteric fat (Table 2; Figure 3B). In subcutaneous fat, exercise increased the number of adipocytes, regardless of the diet (Table 2; Figure 3B). Both exercise and RS lowered the average size of adipocytes in mesenteric fat (Table 2; Figure 3C). Both cell size and number were higher in subcutaneous adipose tissue from EX/SED (C+EX/SED, RS+EX/SED) rats than SED (C, RS) rats (Table 2; Figure 3D), but those on the RS diet had a smaller average diameter than those on the C diet.

### Energy Balance

**Figure 4:**
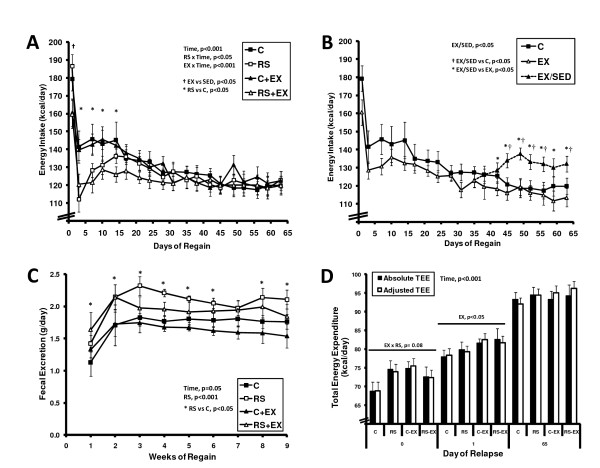
**Energy Intake and Energy Expenditure**. **(A) **Energy intake and **(C) **fecal mass were examined by repeated measures ANOVA for the effect of diet (RS), exercise (EX), time, and their interaction. EXSED rats were excluded from these analyses. **(B) **Energy intake during the final 4 weekd was examined by repeaed measures ANOVA for the effect of EX/SED (C vs EX vs EX/SED) and time. Sedenary RS-fed rats were excluded from this analysis. **(D) **Total Energy Expenditure (TEE) for day 0, 1, and 65 of relapse was examined by repeated measured ANOVA for the effect of diet (RS), exercise (EX), time, and their interaction. Values for EX rats includes the energetic cost of the exercise bout, measured in an exercise indirect calorimetry chamber (~2 kcal/bout). Data is shown before and after the adjusting for the variability in lean mass (p < 0.001) by ANCOVA.

Obese rats consumed 97 ± 1 kcal/d prior to weight loss and 71 ± 1 kCal/d at the end of weight maintenance. Daily exercise expended an additional ~2 kcal/d but did not significantly change the weight maintenance ration of LFD (EX, 72 ± 2, SED 69 ± 2 kCal/d). Exercise attenuated energy intake (EI) by 20 kcal on the first day of relapse (p < 0.01, Figure 4A), but had no significant, independent effect thereafter. In contrast, RS had no effect on EI on the first day of relapse, but attenuated EI on days 3 to 14 (p < 0.05). RS+EX rats experienced both the benefits of exercise (day 1) and RS (days 3-14) on EI. When compared to all other rats, RS+EX rats also ate less from day 7 to day 28 of relapse (126 ± 5 vs 139 ± 3 kcal/d, p < 0.05). Regardless of diet, exercise cessation led to an increase in EI (Figure 4B).

During relapse, fecal mass was 0.3 g/day higher on the RS diet (Figure 4C, p < 0.001). Neither intervention affected the amount of excreted fat (1.1 ± 0.5 kcal/d), but total fecal energy loss was 1.6 kcal/d higher in RS-fed animals.

On day 0, TEE was 72 kcal/d which did not differ between SED and EX rats, despite including the energetic cost of their exercise. No significant difference was observed in TEE with RS or exercise at the end of weight maintenance (day 0), after one day of relapse, or the end of relapse (Figure 4D). However, after adjusting for FM and FFM by ANCOVA, the additional expended energy due to exercise was apparent on day 0 and 1 of relapse (Figure 4D). At the end of the study, TEE of the rats that had stopped exercising was no different from other groups (data not shown).

### Hormones and Metabolites

**Figure 5:**
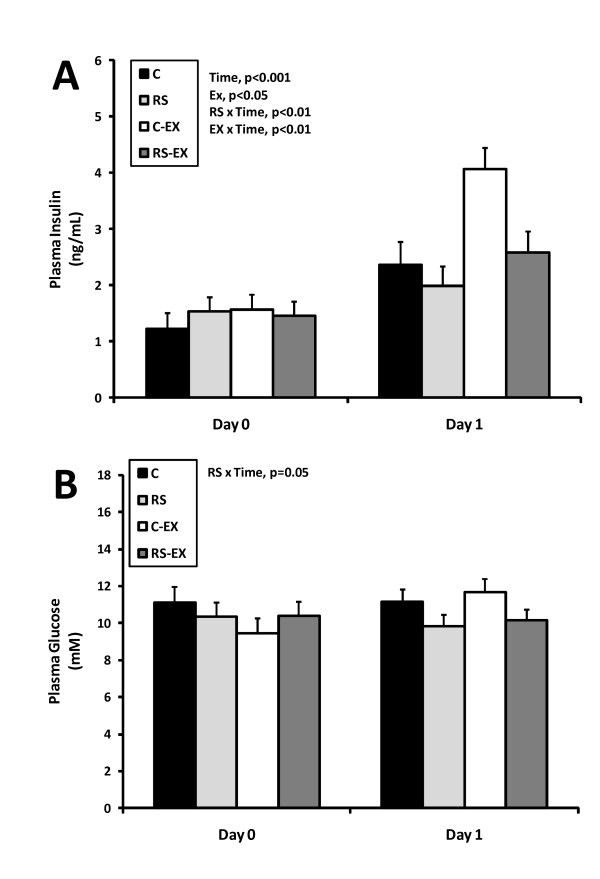
**Plasma Insulin and Glucose at the Transition from Maintenance to Relapse**. Plasma insulin **(A) **and glucose **(B) **on day 0 and day 1 of relapse were examined by repeated measures ANOVA for the effects of diet (RS), exercise (EX), time, and their interaction. Exercise potentiated the increase in insulin on day 1, an effect that was attenuated by dietary RS. Plasma glucose was not affected by time, but on day 1 of relapse glucose levels tended to be lower in RS-fed rats (p = 0.07).

Plasma insulin levels increased after day 1 of relapse on HC/HF diet (p < 0.001). RS blunted this response, whereas exercise enhanced it (Figure 5A). Plasma glucose tended to be lower in RS-fed rats after day 1 (Figure 5B, p = 0.07). At the end of relapse, insulin levels remained lower in RS-fed animals (p < 0.05), with no differences in glucose (data not shown). Other humoral hormones and metabolites are shown in Table 4.

**Table 4:** Endocrine and Metabolite Responses During Relapse.

	C	RS	C+EX	RS+EX
Corticosterone (μg/day) ^C^				
Day 0	3.1 ± 0.3	4.0 ± 0.5	4.4 ± 0.6	4.4 ± 1.1
Day 1	4.0 ± 0.5	4.4 ± 0.4	4.6 ± 0.5	4.6 ± 1.0
Day 65	7.8 ± 1.8	6.9 ± 1.5	6.4 ± 1.6	3.2 ± 1.0 *

Leptin (μg/L) ^C^				
Day 0	2.5 ± 0.6	4.3 ± 1.7	2.8 ± 0.6	2.4 ± 0.4
Day 1	8.9 ± 1.5	8.8 ± 1.5	7.4 ± 1.1	5.9 ± 1.0
Day 65	10.8 ± 2.0	9.4 ± 1.1	8.6 ± 2.1	9.2 ± 1.5

Triglycerides (mmol/L) ^CE^				
Day 0	0.73 ± 0.14	0.64 ± 0.17	0.82 ± 0.16	0.62 ± 0.11
Day 1	2.03 ± 0.15	1.98 ± 0.24	1.87 ± 0.14	1.41 ± 0.28
Day 65	1.62 ± 0.15	1.51 ± 0.24	1.57 ± 0.27	1.13 ± 0.13

NEFA (mmol/L) ^E^				
Day 0	0.54 ± 0.11	0.58 ± 0.11	0.56 ± 0.06	0.52 ± 0.06
Day 1	0.55 ± 0.12	0.46 ± 0.07	0.82 ± 0.16	0.67 ± 0.09
Day 65	0.62 ± 0.10	0.59 ± 0.08	0.67 ± 0.06	0.75 ± 0.07

Cholesterol (mmol/L) ^CD^				
Day 0	1.80 ± 0.09	2.37 ± 0.18 *	2.25 ± 0.23	1.99 ± 0.23
Day 1	2.97 ± 0.28	3.52 ± 0.18	3.94 ± 0.46	2.59 ± 0.28*
Day 65	3.01 ± 0.28	3.48 ± 0.27	4.00 ± 0.57	3.17 ± 0.43

Data were examined by repeated measures ANOVA for the effects of diet (RS), exercise (EX), and time.

EX/SED rats were excluded from this analysis.

All paramaters except NEFAs, were significantly affected by the time of relapse (p < 0.05).

A = RS, p < 0.05; B = EX, p < 0.05; C = time, p < 0.05

D = RS × EX, p < 0.05; E = EX × Time, p < 0.05

* = planned comparison, RS-fed significantly different from corresponding C-fed group, p < 0.05.

Urinary CORT levels (measured over 24 h), a marker of metabolic stress, increased in both EX and SED animals as they regained lost weight (Table 4). Prior to stopping the exercise regimen (week 5), RS+EX/SED rats exhibited lower levels of urinary CORT when compared to C+EX/SED rats (4.7 ± 0.6 vs 2.6 ± 0.2 μg/d, p < 0.05), an effect that was still apparent at the end of relapse (7.7 ± 0.8 vs 2.9 ± 1.4 μg/d, p < 0.05). At the end of the study, CORT was positively associated with total (r = 0.31, p < 0.001) and abdominal (r = 0.40, p < 0·01) fat mass in a combined analysis of all animals.

Plasma cholesterol concentrations were relatively low at day 0 (last day of weight maintenance phase), (Table 4). One day of HC/HF overfeeding induced a dramatic increase in this parameter (p < 0.001) that persisted for the duration of the study.

### Fuel Utilization

**Figure 6:**
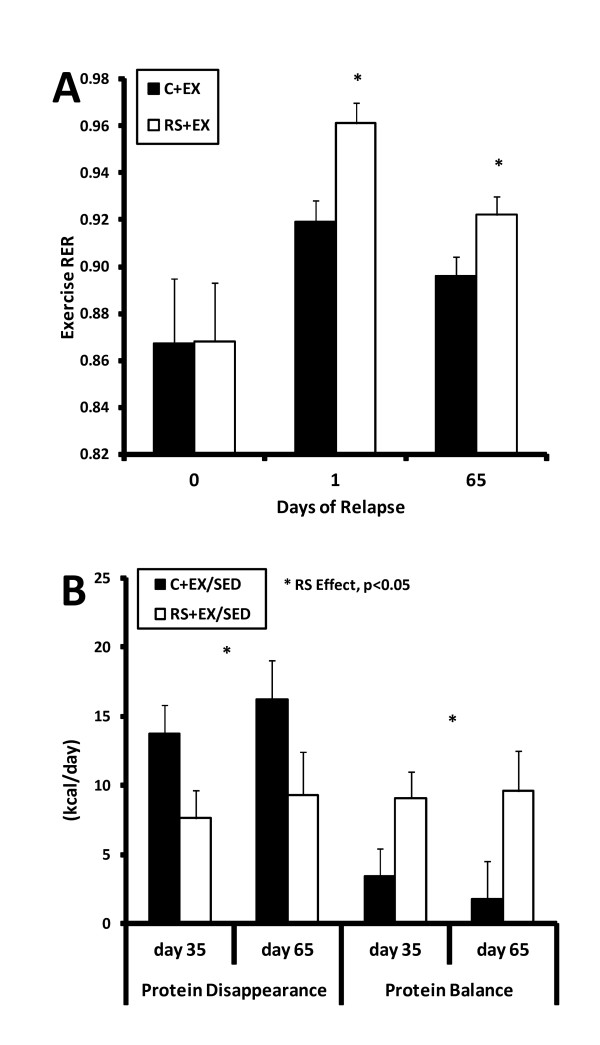
**Fuel Utilization and Protein Balance**. **(A) **The effect of dietary RS on steady-state RER during the exercise bout on days 0, 1 and 65 of relapse is shown. Data on each day were examined by ANCOVA with energy balance as the covariate. Exercise RER was higher in RS-fed rats on day 1 and day 65 of relapse (* p < 0.05), suggesting a greater reliance on carbohydrate, rather than protein or fat, to provide the energy requirements during exercise. **(B) **The effect of dietary RS on protein disappearance and protein balance in EX/SED rats (n = 3/group) shortly before they stopped their program of exercise (day 35 of relapse) and again at the end of the relapse period (day 65 of relapse) is shown. For each parameter, a one-way repeated measure model was used to assess the impact of dietary RS and time. Dietary RS was accompanied by lower protein oxidation and a higher protein balance.

During relapse, steady-state RER during exercise was higher in RS-fed rats compared to C-fed rats (Figure 6A, p < 0.01), suggesting that these animals utilized more carbohydrate in relation to protein and fat during the same absolute workload. In RS+EX/SED rats, protein disappearance (urinary nitrogen), a reflection of protein oxidation, was lower and protein balance was higher at day 35 and 65 of relapse when compared to C+EX/SED rats (Figure 6B).

## Discussion

Novel observations emerged from this study that are relevant to weight regain in humans, but are not likely to emerge from clinical studies. While no intervention prevented weight regain following weight loss, RS and exercise, alone and in combination, attenuated the rate of weight regain and lowered the overall defended body weight. RS and exercise reduced the energy gap between the biological drive to eat and total energy requirements by different mechanisms and at different stages of relapse. The effect of exercise on food intake was at the initial transition to relapse, whereas the effects of RS on food intake occurred during the subsequent weeks. A more subtle effect of their combination on food intake persisted for four weeks after the initial transition from the weight maintenance phase to relapse. Surprisingly, the weight gain prevented by exercise was primarily lean mass, as exercise increased the number of adipocytes in subcutaneous adipose tissue and diverted excess nutrients to this depot. Conversely, RS consumption attenuated regain of fat mass and promoted lean mass accrual when combined with exercise. The distinct mechanisms of these two interventions were clearly complementary in this metabolic context. These observations suggest that RS and exercise, alone or in combination, may facilitate weight maintenance after weight loss by reducing the energy gap between energy intake and expenditure. Their combination may be particularly beneficial during transient excursions from the weight maintenance diet that involve high fat overfeeding.

The magnitude of overfeeding on day 1 of relapse provides insight about the biological pressures to cease or suspend a calorie-restricted, weight maintenance diet. This energy imbalance was roughly twice as large relapsing on a HC/HF diet in comparison to our previous relapse studies with a LF diet [10,11]. Because of the well-known properties of HC/HF diets [34], it was not surprising that the level of overfeeding was greater in the present study than those employing a LF diet. However, the HC/HF diet also greatly reduced the beneficial effects exercise on weight regain. On a LF diet, treadmill exercise reduced the energy gap by ~80% on day 1 of relapse primarily by reducing the magnitude of overfeeding, and this affect on food intake persisted for 8 weeks [12]. Moreover, exercise attenuated fat mass accrual, particularly in abdominal depots, during relapse on a LF diet. The observations in the present study suggest that reverting to unrestricted HC/HF feeding, even for brief periods, may substantially minimize the magnitude and persistence of the beneficial effects of exercise on weight regain and body composition.

In contrast to exercise, the effects of RS on food intake were delayed in initiation but more sustained into the relapse period. This delay may reflect the time for the consumption of RS to affect gut microbiota and the production of short chain fatty acids. The diminution of the RS effect on energy intake occurred as the lost weight was regained, which may reflect a progressive impairment in RS fermentation as the animals relapsed to obesity. In *ad libitum *fed animals, the beneficial effects of RS are dependent upon RS fermentation in the lower gut, and obesity is accompanied by a lack of RS fermentation and no effect of RS on body weight or adiposity [29]. The mechanisms of this obesity-associated lack of RS fermentation are not well understood, but the expectation is that this impairment would return as excess body weight returned. If these observations translate to the human condition, the implication is that RS may be effective for facilitating maintenance of a reduced weight rather than promoting weight loss, *per se*.

RS consumption has been shown to reduce post-prandial excursions in blood glucose and insulin [22,35-39], and these effects translate to the overfeeding event experienced on the first day of relapse. This effect of RS has been attributed to the combination of delayed digestion and fermentation to short chain fatty acids which may improve whole body insulin sensitivity. Insulin and glucose are known to induce the lipogenic pathways [40], and the effects of RS consumption on insulin and glucose are accompanied by reduced expression of adipose tissue glucose transporters (GLUT4) and fatty acid synthase (FAS) and suppression of *de novo *lipogenesis [30,41]. These effects of RS may have contributed to its impact on body composition during relapse in this metabolic context. However, RS, when combined with exercise, prevented the increase CORT excretion that occurred with weight regain, which may also have contributed the preferential deposition of lean mass in this group.

In previous studies with this model, a population of small, presumably new, adipocytes (<20 μm) appears in abdominal adipose depots early in relapse, increasing the total number of adipocytes [10]. The increase in adipocyte number persists to the end of relapse [11,12]. Preferential trafficking of dietary fat and greater retention of lipid from *de novo *lipogenesis in these small adipocytes would not only facilitate clearance of ingested nutrients, but would also contribute to further expansion of the fat depot causing weight gain which surpasses the original, obese weight. Exercise prevents the increase in adipocyte number that results from HF feeding [42] and weight regain on a LFD [12]. Observations in the present study are distinct in that exercise had no effect on cell number in abdominal depots, but instead increased cell number in subcutaneous adipose tissue. Generation of new adipocytes in subcutaneous adipose and the high insulin response in this group may explain the preferential deposition of ingested nutrients in this depot.

It should be noted that, due to its resistance to digestion and passage through the large bowel, the caloric availability of RS is lower than that for digestible starches. Most digestible carbohydrates provide 4kCal/g whereas RS provides around 3 kCal of available energy per g. This difference between the RS and C diets may also be a contributing factor to the lower rate of weight regain and the lower overall defended body weight in the RS rats relative to C rats.

The magnitude of weight regain in this study is greater than that usually observed in clinical studies. Our studies in rodent models reflect the biological pressures to gain weight after weight loss. Unlike rats, humans are subjected to additional psychosocial pressures which motivate them, for reasons unrelated to energy homeostasis, to keep excess weight off. For weight reduced humans, this clearly pits cognitive motivations against the biological drive to regain, with outcomes that can vary greatly in the degree of success. The high recidivism rates observed in obesity therapeutics should provide some warning as to how powerful these biological pressures are. Moreover, clinical studies rarely, if ever, monitor the relapse period in subjects that return to obesogenic dietary habits or follow subjects for more than 5 years [43], and they are limited by a substantial number of drop-outs [44,45]. There is reason suspect that those subjects with the greatest weight gain during follow-up are those who are most reluctant to continue their participation in clinical studies. Regardless, the trajectory of percent weight lost that is regained over 9 weeks in the present study and in our previous studies of weight regain [8,11,12,31] reflect those that occur in humans over 3 to 5 years [1], and, similar to these models, as many as 50% of individuals participating in clinical studies end up surpassing their original weight [45]. Ultimately, the RS diet and exercise still lowered the rate of weight regain and reduced the overall weight regain in this rat model of obesity.

While the EX/SED arm of the present study was not in the initial study design, we have included this data because of the potential implications to the human condition. The inability to sustain a program of regular exercise is a substantial problem in clinical weight loss studies [17] and is a common occurrence in the real world setting. In our LF relapse studies with this model, compliance scores declined sharply at the transition from weight maintenance to relapse [12]. However, they did not progressively decline with weight regain nor were we required to remove any animals from the exercise program. Studies in similar formerly-obese models show that volitional activity also declines after weight loss [15]. These studies suggest that there may be a biological contribution to this noncompliance issue and that it may be exacerbated during relapse on a HC/HF diet.

Because the EX/SED arm was selected based upon compliance scores, rather than randomized, it is unclear if these animals had a pre-existing metabolic disposition, experienced an injury during relapse, or experienced particularly detrimental metabolic effects during the early stages of weight regain. While the EX/SED rats appeared normal at the end of weight maintenance, their metabolic health declined severely by the end of the study. While some beneficial effect of RS was apparent in the protein disappearance and energy balance measures by day 35, it is unclear if the decline in metabolic health occurred early in relapse or after the cessation of exercise. The exercise regimen employed in this study undoubtedly imposed a chronic metabolic stress, and obesity, HF feeding, and overfeeding have been shown to impair the response to chronic stress [46-48]. Exercise cessation may have imparted an additional metabolic insult, as it has known negative consequences on energy balance, weight gain, and metabolic health [49,50]. The impaired ability to respond to these compounding metabolic challenges is likely to have contributed to the detrimental effects of exercise and exercise cessation on body composition. Given the frequent problem with sustained compliance in humans, studies clarifying the impact of exercise cessation after weight loss and during weight regain on a HC/HF diet, beyond the preliminary data reported here, are certainly warranted.

## Conclusions

In summary, our observations indicate that dietary RS, with or without exercise, would be a beneficial component of a weight maintenance strategy following weight loss, even in the face of temporary periods of high fat consumption. The beneficial effects of exercise on weight regain that we have previously reported for relapse on a LF diet appear to be blunted, both in magnitude and duration, when relapse occurs on a HC/HF diet. The complimentary effects of RS and exercise on the biological drive to regain weight may limit overfeeding, reduce the magnitude of regain, and support the accrual of lean mass during transient excursions from a calorie restricted LF diet or the cessation of an exercise program. These findings stress how strong the biological drive to regain lost weight may be and the utility of pursuing a combination of strategies, involving diet, lifestyle, and pharmacotherapy, to counter them. However, our observations suggest that there may be severe metabolic consequences, beyond simple weight regain, of returning to a HC/HF diet and/or a sedentary lifestyle. While diet and lifestyle changes used to achieve weight loss must be sustained permanently to prevent weight regain, it is common that they are not. The underlying reasons for the severe metabolic outcomes in the EX/SED arm of this study need to be clarified and their implications, if any, for weight regain in humans need to be considered. Given the data presented here, relapse of the formerly-obese, even to the former weight, under high fat conditions is likely to be accompanied by detrimental changes in body composition leaving the patient at greater risk for co-morbidities.

## Competing interests

The authors declare that they have no competing interests.

## Authors' contributions

All authors read and approved the final manuscript. JAH prepared the manuscript, assisted in the planning and conduct of all experiments, and data interpretation; ILB, assisted in the planning of experiments, data interpretation, and manuscript preparation; HRW and JOH assisted in the planning of experiments; GCJ and AS conducted experiments, assays, and data entry; MRJ assisted in the planning and conduct of experiments and assays, data interpretation, and manuscript preparation; PSM prepared the manuscript and assisted in the planning and conduct of experiments, data entry, data interpretation, and statistical analysis.
